# Development and validation of simplified prognostic models for 14- and 30-day mortality in advanced cancer: beyond the glasgow prognostic score

**DOI:** 10.3389/fonc.2026.1729131

**Published:** 2026-02-05

**Authors:** Jhen-Ling Huang, Wan-Ling Yang

**Affiliations:** 1 Far Eastern Memorial Hospital, Taipei, Taiwan; 2 Department of Nursing, Taipei Veterans General Hospital, Taipei, Taiwan

**Keywords:** advanced cancer, decision-curve analysis, external validation, Glasgow Prognostic Score, modified GPS, palliative care, prognostication, short-term mortality

## Abstract

**Background:**

Reliable week-level survival prediction is needed for hospice referral and end-of-life decisions in advanced cancer. The Glasgow Prognostic Score (GPS) and modified GPS (mGPS) are widely used for long-term outcomes, but their short-term value is uncertain.

**Methods:**

In a retrospective cohort of 6,063 patients with advanced cancer from a single center (5,315 development; 748 external validation), we evaluated GPS/mGPS for 14- and 30-day mortality and developed two logistic-regression models using routinely available variables: an L1-regularized “full” model and a simplified eight-variable model selected via least absolute shrinkage and selection operator. Performance was assessed by area under the receiver operating characteristic curve (AUC), Brier score, calibration, and decision-curve analysis.

**Results:**

GPS and mGPS demonstrated poor discrimination and suboptimal calibration for short-term mortality (AUC 0.52–0.55). In contrast, the full model improved discrimination for 14- and 30-day mortality (AUCs 0.663 and 0.654, respectively), with lower Brier scores and better calibration. The simplified model achieved comparable performance (AUCs 0.652 and 0.636). Both models provided modest net clinical benefit across clinically relevant threshold probabilities.

**Conclusion:**

GPS and mGPS are inadequate for week-level prognostication in advanced cancer, whereas simplified models integrating objective clinical and laboratory variables improve discrimination, calibration, and potential clinical utility. The eight-variable model may facilitate real-world implementation in end-of-life care.

## Introduction

Prognostication of survival in patients with advanced cancer is a core component of palliative care. Although clinician estimates are frequently inaccurate and often overly optimistic, a range of clinical, biological, and symptom-based factors—as well as validated prognostic models—can improve short-term survival predictions. Such prognostic information is essential for guiding treatment decisions, timely hospice referral, and effective communication with patients and families (1–3). Unlike long-term prognostication, where survival is measured in months, end-of-life care often requires clinicians to predict survival within weeks, a task that remains clinically challenging.

Several prognostic tools have been developed to address this need, including the Palliative Prognostic Score (PaP) (4), Palliative Prognostic Index (PPI) (5), Palliative Performance Scale (PPS) (6), and the Prognosis in Palliative Care Study (PiPS) models (7). While these instruments combine performance status, symptoms, and laboratory data to achieve reasonable accuracy, their reliance on subjective judgments and multiple input variables can limit feasibility in routine or resource-limited practice (2, 3).

The Glasgow Prognostic Score (GPS), derived solely from C-reactive protein (CRP) and albumin, provides an objective and readily available alternative (8). The modified Glasgow Prognostic Score (mGPS) represents a refinement of the original inflammation-based approach, addressing some of its limitations and broadening its clinical utility (9). Over time, it has moved from the research arena into mainstream care, now finding its place in international palliative care guidelines (10). GPS has been validated in many cancer populations for long-term outcomes (9), yet its role in short-term 14- and 30-day survival prediction has not been systematically examined. This evidence gap is particularly relevant for end-of-life decision-making.

Therefore, this study aimed to evaluate the performance of GPS and mGPS in predicting imminent mortality, while also developing and validating new models that integrate symptoms, comorbidities, and laboratory markers to improve clinical applicability.

## Materials and methods

### Study population

**Figure 1:**
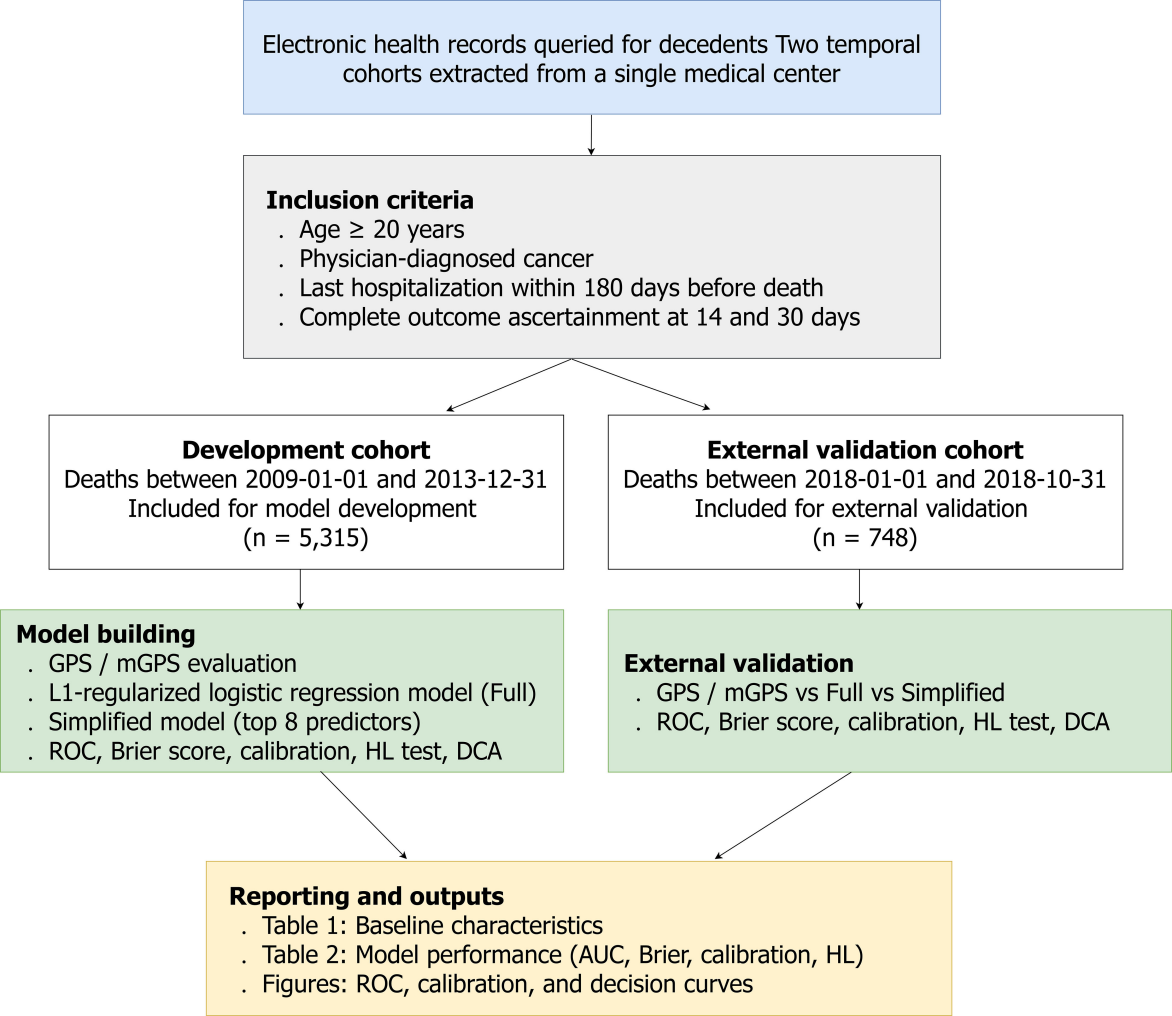
Study flowchart showing cohort construction, inclusion criteria, and analytic workflow.

This retrospective cohort study was conducted at Taipei Veterans General Hospital using a death-based design of adult patients with cancer at an advanced/end-of-life stage. The study flow diagram is shown in **Figure 1**. Rather than relying on formal clinical tumor staging, the study focused on predicting short-term post-discharge mortality following the last hospitalization, with mortality assessed at 14 and 30 days after discharge, as staging information (e.g., TNM stage) was not consistently available in the electronic health record.

For model development, we identified 5,315 cancer decedents who died between January 1, 2009 and December 31, 2013. For external validation, we included an independent cohort of 748 cancer decedents who died between January 1, 2018 and October 31, 2018. Eligible patients were ≥20 years of age, had a physician-documented diagnosis of cancer, and had a last hospitalization within 180 days before death. The index time point (time zero) was defined as discharge from the last hospitalization, and outcomes were defined as death within 14 and 30 days after discharge. Information on active systemic anticancer treatment and hospitalization attribution (e.g., treatment-related toxicity vs disease progression) was not consistently available; therefore, we could not reliably distinguish the primary reason for hospitalization.

Lymphoma was the only hematologic malignancy included in this study and was analyzed as a distinct cancer-type category (reported in **Table 1**).

**Table 1:** Baseline characteristics of the development and validation cohorts.

Variable	Development (n=5,315)	Validation (n=748)	P-value
Age, years	68.9 ± 14.9	68.0 ± 13.8	0.101
Length of stay, days	21.0 ± 21.8	22.8 ± 21.1	0.024
CCI	8.51 ± 0.86	8.52 ± 0.90	0.761
Male, n (%)	1,775 (33.4)	296 (39.6)	<0.001
Admission via ER, n (%)	2,857 (53.8)	470 (62.8)	<0.001
Lung cancer, n (%)	1,452 (27.3)	184 (24.6)	0.117
Liver cancer, n (%)	820 (15.4)	125 (16.7)	0.365
Colorectal cancer, n (%)	604 (11.4)	92 (12.3)	0.452
Gastric cancer, n (%)	441 (8.3)	44 (5.9)	0.023
Lymphoma, n (%)	322 (6.1)	37 (4.9)	0.228
Dyspnea, n (%)	1062 (20.0)	159 (21.3)	0.415
Pain, n (%)	2,159 (40.6)	291 (38.9)	0.370
Fever, n (%)	1,034 (19.5)	157 (21.0)	0.323
Edema, n (%)	665 (12.5)	119 (15.9)	0.010
Jaundice, n (%)	328 (6.2)	35 (4.7)	0.107
Pleural effusion, n (%)	1,236 (23.3)	165 (22.1)	0.467
Ascites, n (%)	543 (10.2)	81 (10.8)	0.606
Diarrhea, n (%)	635 (11.9)	106 (14.2)	0.082
Nausea vomiting, n (%)	993 (18.7)	160 (21.4)	0.077
Anorexia, n (%)	1,354 (25.5)	203 (27.1)	0.329
Fatigue, n (%)	120 (2.3)	36 (4.8)	<0.001
Cachexia, n (%)	69 (1.3)	16 (2.1)	0.067
Weakness, n (%)	835 (15.7)	124 (16.6)	0.543
Insomnia, n (%)	127 (2.4)	22 (2.9)	0.362
Constipation, n (%)	356 (6.7)	62 (8.3)	0.108
Depression, n (%)	82 (1.5)	8 (1.1)	0.316
Weight loss, n (%)	501 (9.4)	59 (7.9)	0.174
Consciousness disturbance, n (%)	149 (2.8)	19 (2.5)	0.681
Delirium, n (%)	138 (2.6)	18 (2.4)	0.759
Infection, n (%)	1,054 (19.8)	161 (21.5)	0.279
Leukocytosis, n (%)	121 (2.3)	23 (3.1)	0.179
Leukopenia, n (%)	91 (1.7)	18 (2.4)	0.181
Dysphagia, n (%)	150 (2.8)	20 (2.7)	0.818

Data are presented as mean ± SD or n (%). P-values are derived from Welch’s t-test for continuous variables and chi-square or Fisher’s exact test for categorical variables.

### Ethics approval

This study was approved by the Institutional Review Board of Taipei Veterans General Hospital (IRB No 2018-07-023AC). Informed consent was waived due to the retrospective design. The study adhered to the Declaration of Helsinki and Good Clinical Practice guidelines.

### Data collection and processing

Clinical data were retrieved from the institutional electronic health record system, including both structured and unstructured data. Structured data were extracted via Microsoft SQL Server, whereas unstructured admission notes were processed using Amazon Comprehend Medical for natural language processing. All records were de-identified before analysis. Fever and infection were identified based on broad clinical documentation and structured diagnosis codes; febrile neutropenia could not be reliably ascertained because absolute neutrophil counts at the time of fever onset were not consistently available.

Missing laboratory values were imputed using multiple imputation. Extracted variables included demographics (age, sex, emergency admission, Charlson Comorbidity Index, length of stay), cancer type (14 categories), 23 binary symptoms (e.g., dyspnea, pain, fever, edema, jaundice, pleural effusion, ascites, anorexia, fatigue, cachexia), and laboratory measures (WBC, hemoglobin, platelets, lymphocyte %, ALKP, ALT, AST, bilirubin, BUN, creatinine, calcium, potassium, sodium, albumin, CRP, LDH).

Hospital admissions were clinically heterogeneous, but active systemic treatment status and admission attribution (treatment-related toxicity vs disease progression) were not reliably available; therefore, these could not be distinguished in this dataset. This limitation was considered in the interpretation of the results.

### Statistical analysis

Baseline characteristics were summarized as mean ± standard deviation (SD) for continuous variables and as number (percentage) for categorical variables. Comparisons between the development and validation cohorts were performed using Welch’s t-test for continuous variables and chi-square or Fisher’s exact test, as appropriate, for categorical variables.

For prognostic model evaluation, we examined discrimination, calibration, and overall performance. Discrimination was quantified using the area under the receiver operating characteristic curve (AUC). Overall accuracy was assessed with the Brier score. Calibration was evaluated using logistic regression–based calibration intercepts and slopes, calibration plots, and the Hosmer–Lemeshow goodness-of-fit test. Clinical utility was assessed through decision curve analysis (DCA).

We first evaluated the Glasgow Prognostic Score (GPS) and modified GPS (mGPS) for predicting 14- and 30-day mortality. Analyses for 21-day mortality were also conducted and are reported in the **Supplementary Materials**, whereas the main text focuses on 14- and 30-day outcomes for clarity and conciseness. To explore potential improvements, we then developed two new models: (1) a full L1-regularized logistic regression model including all candidate variables, and (2) a simplified model including the top predictors selected by LASSO to enhance clinical usability. The eight variables selected by LASSO for 30-day mortality are provided in **Supplementary Table 1**.

Kaplan–Meier survival curves, stratified by predicted 30-day mortality risk derived from the simplified LASSO model, were generated for illustrative purposes (**Supplementary Figure 1**). Patients were categorized into low-, intermediate-, and high-risk groups based on tertiles of the predicted 30-day mortality probability.

All statistical analyses were conducted in Python (version 3.10) within Google Colaboratory (Google LLC, Mountain View, CA, USA). Key libraries included pandas (v1.5.3) for data processing, scikit-learn (v1.2.2) for model development and validation, statsmodels (v0.13.5) for regression and calibration analyses, and matplotlib (v3.7.1) and seaborn (v0.12.2) for data visualization. A two-sided p < 0.05 was considered statistically significant.

## Results

### Baseline characteristics

A total of 6,063 patients with advanced cancer were included, comprising 5,315 patients in the development cohort and 748 patients in the external validation cohort. The mean age was similar between cohorts (68.9 ± 14.9 years in the development cohort vs. 68.0 ± 13.8 years in the validation cohort). The proportion of male patients was higher in the validation cohort (39.6% vs. 33.4%, *p* < 0.001). Emergency admission was also more frequent in the validation cohort (62.8% vs. 53.8%, *p* < 0.001). With respect to cancer types, gastric cancer was less frequent in the validation cohort (5.9% vs. 8.3%, *p* = 0.023), whereas the distribution of other malignancies was broadly comparable across cohorts. Several symptoms were more prevalent in the validation cohort, reflecting potential temporal or population differences. Full baseline characteristics are presented in **Table 1**.

### Performance of GPS and mGPS

**Figure 2:**
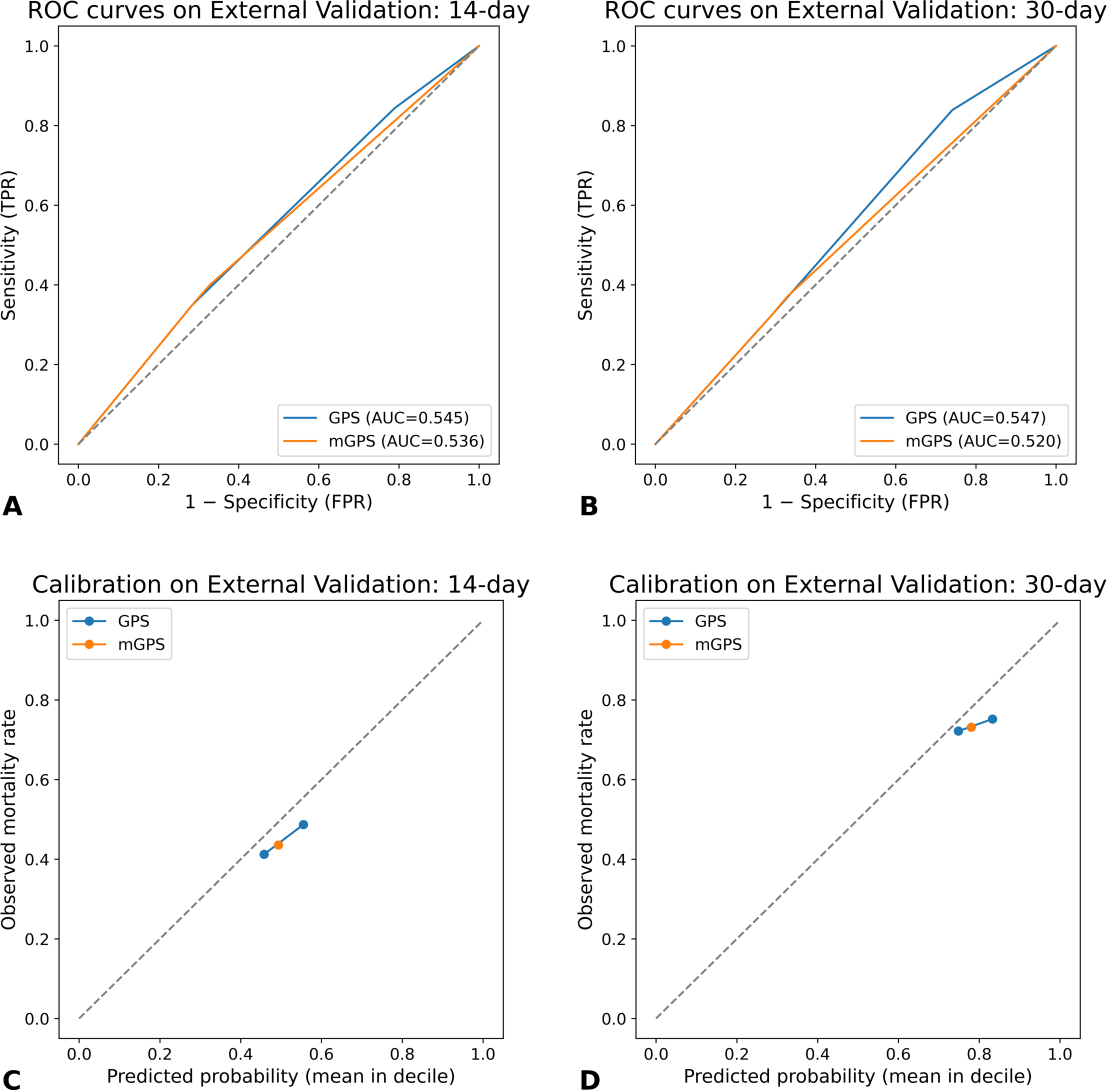
External validation of GPS and mGPS for short-term mortality. **(A)** Receiver operating characteristic (ROC) curves for 14-day mortality. **(B)** ROC curves for 30-day mortality. **(C)** Calibration plot for 14-day mortality. **(D)** Calibration plot for 30-day mortality.

The discriminative performance of the Glasgow Prognostic Score (GPS) and modified GPS (mGPS) for predicting short-term mortality was limited. Across the 14- and 30-day horizons, AUC values ranged from 0.52 to 0.55 (**Figures 2A, B**), indicating performance only marginally better than chance. Calibration analyses demonstrated slopes deviating considerably from 1, suggesting poor alignment between predicted probabilities and observed mortality. Although Hosmer–Lemeshow (HL) test *p*-values were >0.05 in most analyses, indicating no formal statistical evidence of miscalibration, the graphical calibration plots (**Figures 2C, D**) demonstrated that both GPS and mGPS tended to underestimate risk in higher-risk deciles and overestimate risk in lower-risk groups. Collectively, these results suggest that GPS-based scoring systems have limited utility for short-term prognostication in patients with advanced cancer.

### Development and validation of new models

**Figure 3:**
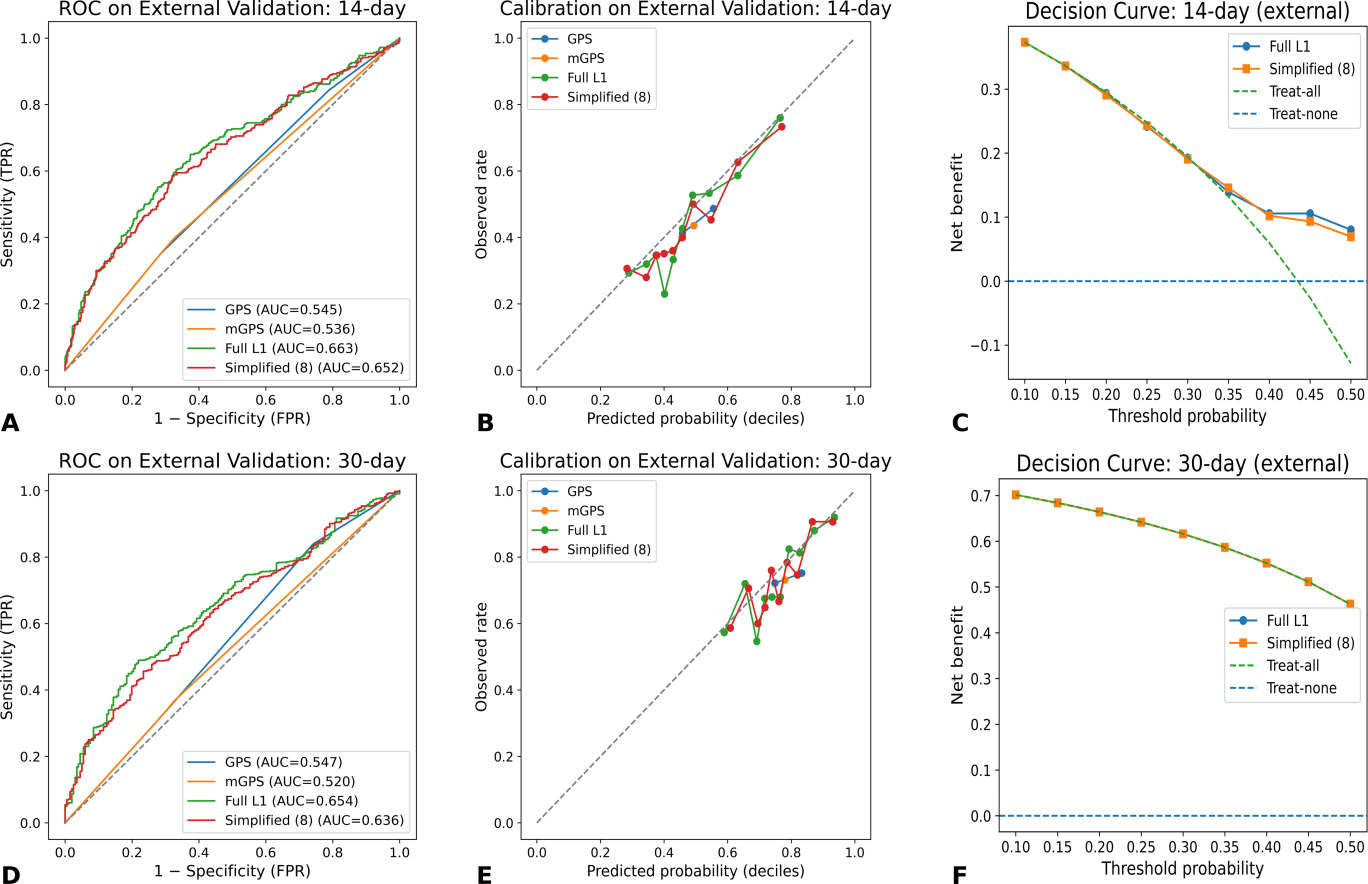
Performance of newly developed models compared with simplified versions in external validation. **(A)** ROC curves for 14-day mortality. **(B)** Calibration plot for 14-day mortality. **(C)** Decision curve analysis for 14-day mortality. **(D)** ROC curves for 30-day mortality. **(E)** Calibration plot for 30-day mortality. **(F)** Decision curve analysis for 30-day mortality.

To address these limitations, we developed two new models. The full L1-regularized logistic regression model incorporated all candidate variables, including demographics, comorbidities, symptoms, and laboratory parameters. This model achieved AUCs of 0.663 and 0.654 for 14- and 30-day mortality, respectively, representing an improvement of approximately 10–12 percentage points compared with GPS and mGPS. The simplified model, which retained only the eight most predictive variables selected by LASSO regression, demonstrated comparable performance, with AUCs of 0.652 and 0.636 across the two horizons (**Figures 3A, D**).

Brier scores further confirmed the superior accuracy of the new models. For example, Brier scores for GPS/mGPS ranged from 0.197 to 0.248, whereas the full and simplified models consistently achieved lower values between 0.187 and 0.229, reflecting improved overall probability estimates. Calibration analyses also favored the new models. As shown in (**Figures 3B, E**), the calibration curves of both full and simplified models were closer to the ideal 45° line compared with GPS/mGPS. Calibration slopes for the full L1 model approached unity (0.850–0.990), with intercepts close to zero, indicating good calibration. The simplified model achieved similar results, underscoring that robust performance can be maintained even with a reduced set of variables. Detailed metrics, including AUCs, Brier scores, calibration slopes, and HL test results, are summarized in **Table 2**.

**Table 2:** Model performance across 14- and 30-day mortality prediction horizons.

Horizon	Model	AUC	Brier	Intercept	Slope	HL χ²	HL p-value	Prevalence
14-day	GPS	0.545	0.247	-0.222	0.808	8.725	0.366	0.436
	mGPS	0.536	0.248	-0.230	1.135	9.970	0.267	0.436
	Full L1	0.663	0.226	-0.153	1.068	13.737	0.089	0.436
	Simplified (8)	0.652	0.229	-0.169	0.950	8.269	0.408	0.436
30-day	GPS	0.547	0.197	0.158	0.676	12.982	0.112	0.731
	mGPS	0.520	0.199	0.354	0.509	10.466	0.234	0.731
	Full L1	0.654	0.187	-0.141	0.990	14.712	0.065	0.731
	Simplified (8)	0.636	0.188	-0.120	0.970	13.913	0.084	0.731

AUC, area under the ROC curve; HL, Hosmer–Lemeshow test. Variables were ranked by absolute coefficient size in the Simplified model.

### Decision curve analysis

Clinical utility was evaluated using decision curve analysis (DCA). As shown in (**Figures 3C, F**), GPS and mGPS offered minimal net benefit across clinically relevant threshold probabilities. In contrast, both the full and simplified models provided modest but consistent net benefit, particularly at the 30-day horizon. For instance, when threshold probabilities ranged from 0.2 to 0.4, the full and simplified models demonstrated higher net benefit compared with treat-all or treat-none strategies, suggesting potential added value for guiding decisions such as hospice referral or initiation of palliative interventions. Although the absolute magnitude of benefit was modest, these gains may still be clinically meaningful in the context of end-of-life care, where even small improvements in prognostic accuracy can influence patient and family decision-making.

### Summary of findings

Overall, GPS and mGPS alone demonstrated poor discriminative and calibration performance for short-term mortality prediction, limiting their clinical applicability. In contrast, both the full L1 model and the simplified eight-variable model showed improved accuracy, better calibration, and modest net clinical benefit. Importantly, the simplified model preserved predictive performance while requiring only a limited set of routinely available variables, suggesting greater feasibility for real-world implementation. These findings highlight the potential of integrative, data-driven models to provide more accurate and clinically useful short-term prognostication in advanced cancer.

## Discussion

### Principal findings

In this retrospective cohort, GPS and mGPS demonstrated limited discriminative performance for 14- and 30-day mortality, with AUCs approximating random chance. These findings reinforce earlier observations that inflammation-based indices, though useful for long-term prognosis, are inadequate for imminent survival prediction (1, 8–10).

To address this limitation, we developed two alternative models. The full L1-regularized model integrating demographics, symptoms, comorbidities, and laboratory variables achieved higher discrimination and better calibration at both 14- and 30-day horizons. Notably, a simplified eight-variable version preserved much of this predictive ability, offering a pragmatic tool suitable for bedside use or electronic health record integration. Both models also showed modest gains in decision curve analysis, suggesting incremental clinical utility when applied alongside physician judgment (2, 11).

### Interpretation of model performance

The poor performance of GPS and mGPS in our study is not unexpected, given their reliance on only two laboratory values: C-reactive protein and albumin. Although systemic inflammation is an established marker of long-term outcomes (8–10), its temporal fluctuations and limited sensitivity to imminent physiologic decline make it unsuitable for predicting death within weeks. Our results reinforce the limitations reported in prior meta-analyses, where the predictive accuracy of inflammation-based indices was markedly lower for short-term endpoints compared with longer horizons (3, 10).

By contrast, our full L1 model incorporated a broad set of demographic, comorbidity, symptom, and biochemical variables, yielding relative improvements of approximately 10 percentage points in AUC over GPS/mGPS. In addition to improved discrimination, the new models achieved lower Brier scores, indicating more accurate probability estimates. Calibration slopes close to 1 and intercepts near 0 further suggest that risk predictions from the new models are more clinically trustworthy than those generated by GPS or mGPS. Importantly, well-calibrated models provide clinicians with estimates that can be more confidently communicated to patients and families, thereby supporting shared decision-making in palliative care (1). These improvements are clinically relevant: even modest gains in discrimination and calibration may support better timing of hospice referral, initiation of comfort-focused care, and communication with families when combined with physician judgment (2).

Equally important, the simplified eight-variable model preserved much of the predictive ability of the full model, demonstrating that parsimonious predictor sets can achieve clinically meaningful accuracy. This is particularly valuable in palliative care settings, where feasibility, ease of use, and minimal burden on clinicians are essential. While calibration performance varied modestly across time horizons, including supplementary analyses, the overall calibration of both models was acceptable. This balance of accuracy and simplicity suggests that simplified models may represent a practical compromise between statistical rigor and bedside usability.

It should be noted that our definition of advanced cancer was based on short-term mortality following the last hospitalization, rather than formal clinical staging. This death-based operational definition reflects real-world end-of-life trajectories captured in retrospective data, but may also introduce heterogeneity in disease status at the time of admission. The moderate discrimination observed in our models likely reflects the inherent difficulty of week-level prognostication in advanced cancer, where clinical trajectories are often nonlinear and influenced by acute events. In addition, heterogeneity in tumor biology, treatment exposure, and reasons for hospitalization may further limit achievable discrimination.

Differences observed between the development cohort (2009–2013) and the external validation cohort (2018) likely reflect temporal changes in oncology practice, supportive care, and inpatient management over time. Importantly, validation in a temporally distinct cohort provides a more stringent assessment of model robustness and supports the applicability of the models across evolving clinical contexts. In theory, restricting analyses to more homogeneous subgroups—such as patients with advanced solid tumors and hospitalizations unrelated to treatment-related toxicity—could improve model performance by reducing clinical variability. However, such restrictions would also reduce sample size and limit real-world applicability, particularly in inpatient settings where prognostic uncertainty is greatest.

### Comparison with previous studies

Our results are consistent with prior research showing that PaP, PPI, PPS, and PiPS models generally outperform GPS for short-term prognostication (4–7, 10, 12). While inflammation-based scores remain attractive for their simplicity, their predictive accuracy is limited when week-level estimates are needed (3, 8, 10). By contrast, integrative models that include symptom burden and biochemical markers better capture the multidimensional decline of patients near the end of life (1, 10).

Recently developed tools such as PROMISE have demonstrated strong prognostic performance in oncology populations receiving active anticancer treatment. This is particularly evident when detailed treatment response and imaging data are available, as shown in recent development and validation studies (13, 14). However, direct comparison with PROMISE was not feasible in the present study, as systematic information on radiologic treatment response and best response classification was not consistently available in our retrospective, death-based cohort focused on the last hospitalization. In addition, PROMISE was primarily developed for patients undergoing ongoing systemic therapy, whereas our study population consisted of hospitalized patients near the end of life, many of whom may not have been receiving active treatment. Future studies directly comparing simplified inpatient prognostic models with PROMISE in datasets containing comprehensive treatment and imaging information would be valuable.

Furthermore, prior validations of PaP and PiPS in diverse palliative populations have reported AUCs in the range of 0.65–0.72 (10, 15), which is similar to the performance observed for our full and simplified models. This suggests that incorporating both objective laboratory data and patient-centered variables yields the most reliable short-term prognostic estimates. Unlike some existing tools that require physician-rated performance status, our simplified model relies entirely on routinely collected clinical and laboratory data, which may enhance reproducibility and reduce inter-rater variability (7, 10, 15). In this regard, our findings extend prior work by showing that meaningful prognostic performance can be achieved even without subjective scoring, a feature that may improve clinical uptake in busy hospital settings.

### Clinical implications

These findings have several practical implications. First, they caution against reliance on GPS or mGPS alone for short-term prognostication. In practice, using these indices could lead to misleadingly optimistic or pessimistic survival estimates, potentially resulting in delayed hospice referral, misaligned treatment decisions, or missed opportunities for timely family discussions. Second, the simplified eight-variable model we developed may offer a feasible alternative that balances predictive accuracy with usability. Because the predictors are objective, routinely collected, and available in most hospital information systems, the model is amenable to real-world implementation, including integration into electronic health records.

Third, although the absolute gains in AUC and Brier score were modest, even small improvements in predictive accuracy can be valuable in the end-of-life context. Prognostication in terminal illness is inherently uncertain (1, 2), and tools that enhance accuracy and calibration, even incrementally, can support clinicians in making more confident recommendations. When combined with physician judgment, structured prognostic models may reduce variation in clinical practice, facilitate earlier palliative care referral, and improve alignment of care with patient goals (10, 11). Finally, the demonstration that a reduced set of eight predictors retains strong performance suggests that prognostic modeling need not be overly complex to provide clinical value and that future models may further streamline input requirements. From a practical standpoint, this model is intended to be applied at the time of hospitalization or early during inpatient care, when routinely collected clinical, symptom, and laboratory data are readily available. In this setting, the model may support clinicians in identifying patients at high risk of near-term mortality and prompt timely discussions regarding goals of care, hospice referral, or escalation of palliative interventions.

### Strengths and limitations

Our study has notable strengths. It included a large development cohort and a temporally distinct external validation cohort, enhancing the robustness and generalizability of findings. The use of natural language processing to extract symptom data from unstructured records reduced reliance on manual chart review, while multiple imputation for missing laboratory values minimized bias. This comprehensive evaluation of discrimination, calibration, and clinical utility, rather than focusing solely on AUC, provides a more nuanced assessment of model performance.

Nevertheless, several limitations should be acknowledged. First, this was a single-center study conducted in Taiwan, which may limit generalizability to other populations and healthcare systems.

Second, the tumor type distribution in our cohort (**Table 1**) may reflect potential selection bias. Cancers with relatively indolent disease trajectories, such as breast and prostate cancer, and other epidemiologically common cancers were underrepresented compared with their epidemiological prevalence. This likely reflects the death-based, hospitalization-focused study design, as patients with these tumor types are less likely to require inpatient admission near the end of life or to die shortly after hospitalization. Consequently, the generalizability of our findings may be greater for patients with aggressive solid tumors and advanced disease requiring inpatient care, and caution is warranted when extrapolating the results to cancer populations with slower disease courses.

Third, formal clinical cancer staging and detailed treatment information were not consistently available in the electronic health record. As a result, we could not determine whether patients were receiving active systemic anticancer therapy at the time of hospitalization, nor could we reliably distinguish admissions related to treatment-related toxicity from those due to disease progression. This heterogeneity may have introduced variability in short-term mortality risk and model performance. In addition, a substantial proportion of hospitalizations were classified as non-emergent admissions, which in this context may represent planned or semi-urgent inpatient care for symptom control, management of advanced cancer–related complications, or transitions in goals of care rather than clinical stability. Retaining these admissions reflects real-world end-of-life care pathways but may further contribute to clinical heterogeneity. Febrile neutropenia could not be reliably identified as a distinct clinical entity in the electronic health record (e.g., absolute neutrophil counts at fever onset were not consistently available); therefore, cases of febrile neutropenia may have been included within the broader categories of fever or infection and may have acted as a confounding factor in short-term mortality prediction.

Fourth, some established prognostic factors, such as performance status or imaging biomarkers, were not available in our dataset and could have further improved prediction. In addition, laboratory measures were extracted during the index hospitalization (defined as the last hospitalization within 180 days before death). While this approach reflects routinely available inpatient data at the time of clinical decision-making, it may still allow for temporal variability and may not always capture the most acute physiological derangements immediately preceding death. This limitation may have attenuated short-term prognostic accuracy and should be considered when interpreting the results.

Finally, the retrospective design precludes direct evaluation of how these models would influence clinical decision making in real time. Prospective validation and integration into care pathways will be critical next steps.

## Conclusion of discussion

Taken together, these findings highlight the limited value of GPS and mGPS for short-term prognostication in advanced cancer and underscore the advantages of integrative models that combine symptom and laboratory information. The simplified eight-variable model offers a pragmatic balance between accuracy and feasibility, with potential applicability in real-world clinical settings. These results underscore the importance of moving beyond inflammation-based indices toward multidimensional models that better capture the complexity of end-of-life decline. Future studies with reliably captured treatment exposure and admission attribution (toxicity vs progression) could formally evaluate whether restricting analyses to more homogeneous inpatient subgroups improves discrimination for week-level mortality prediction.

## Data Availability

The raw, anonymized data supporting the conclusions of this article will be made available by the authors upon reasonable request, in compliance with ethical and privacy regulations. Requests to access the datasets should be directed to Wan-Ling Yang, wlyang@vghtpe.gov.tw.
