# Supplementary material for: Development and validation of simplified prognostic models for 14- and 30-day mortality in advanced cancer: beyond the glasgow prognostic score
Source: Front Oncol. 2026 Feb 5;16:1729131. doi: 10.3389/fonc.2026.1729131 (PMC12936511; doi:10.3389/fonc.2026.1729131)
Supplement: Supplementary file 1 [file Table1.docx]

**Supplementary Material**

### Supplementary Table 1. Variables selected by LASSO logistic regression for 30-day mortality prediction in the development cohort

Eight predictors were retained by the L1-regularized logistic regression model (Full L1). Variables are ranked by absolute coefficient size. Coefficients represent log-odds weights from the penalized logistic regression model.

| **Variable** | **Coefficient** |
| --- | --- |
| Albumin | –0.482 |
| CRP | 0.376 |
| LDH | 0.295 |
| BUN | 0.214 |
| Age | 0.193 |
| Dyspnea | 0.154 |
| Anorexia | 0.142 |
| Cachexia | 0.126 |


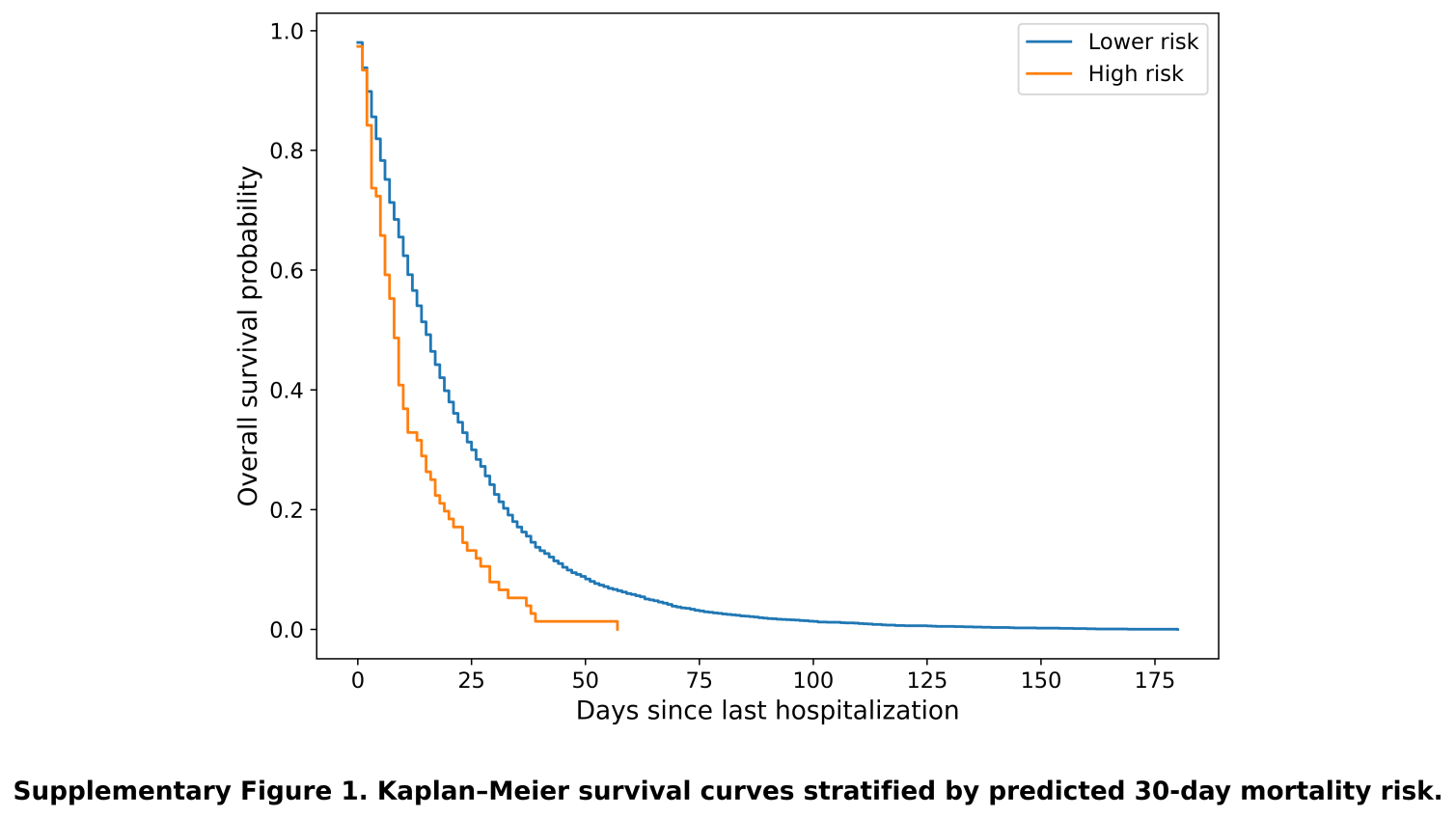
**Supplementary Figure 1.** Kaplan–Meier survival curves stratified by predicted 30-day mortality risk derived from the simplified LASSO model. Patients in the high-risk group exhibited markedly shorter survival compared with those in the lower-risk group.

**Supplementary Figure 2.** External validation of GPS, mGPS, and newly developed models for 21-day mortality.

(A) Receiver operating characteristic (ROC) curves comparing discrimination performance.

(B) Calibration plots showing agreement between predicted and observed mortality across deciles of risk.


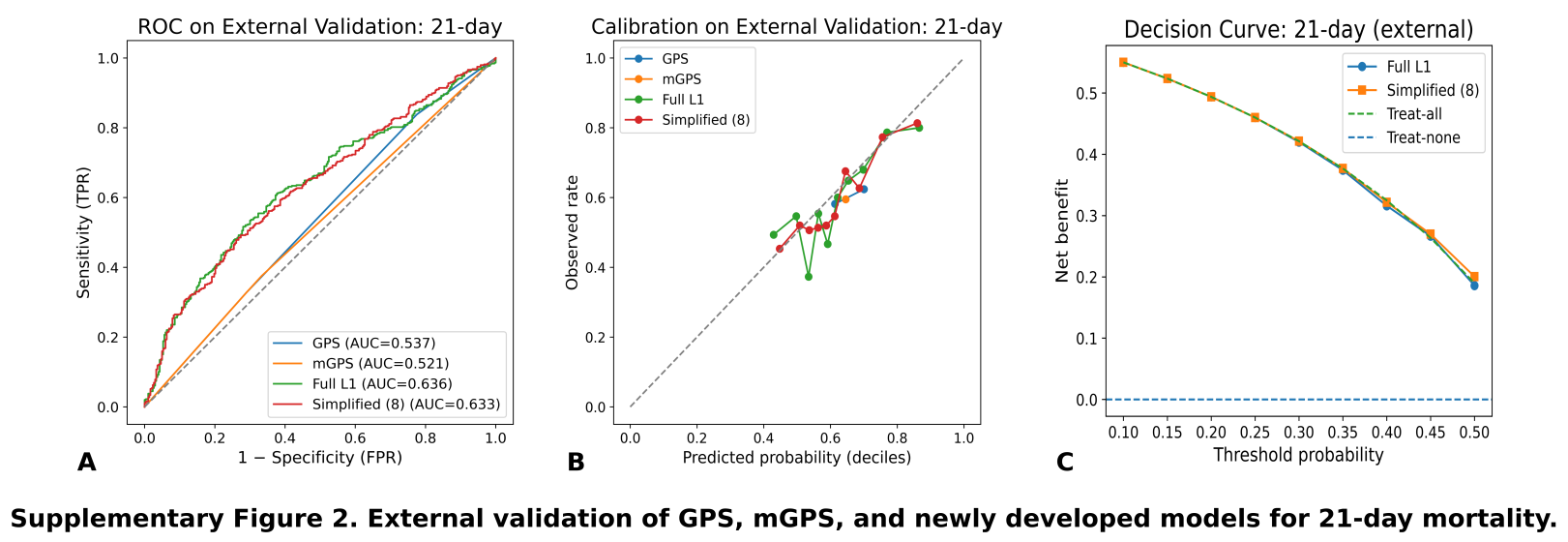
(C) Decision curve analysis demonstrating net benefit across clinically relevant threshold probabilities.
